# Effects of Maternal Tetramethyl Bisphenol F Exposure on Neurodevelopment and Behavior in Mouse Offspring

**DOI:** 10.3390/ijms27073299

**Published:** 2026-04-05

**Authors:** Inho Hwang, Sun Kim, Eui-Bae Jeung

**Affiliations:** Department of Veterinary Medicine, College of Veterinary Medicine, Chungbuk National University, Cheongju 28644, Republic of Korea; darkpower777@nate.com (I.H.); boksagi88@gmail.com (S.K.)

**Keywords:** tetramethyl bisphenol F, Developmental neurotoxicity, maternal exposure, Sox1−GFP embryonic stem cells, behavioral impairment, synaptic plasticity, endocrine-disrupting chemicals

## Abstract

Bisphenol A (BPA) has long been used in plastics, resins, and food packaging materials; however, extensive research has demonstrated its reproductive, developmental, and endocrine-disrupting effects. Consequently, BPA has been increasingly restricted and replaced with structural analogues. Among these, tetramethyl bisphenol F (TMBPF) has emerged as one of the most widely used substitutes, particularly in epoxy resins and food-can coatings. Although initially regarded as a safer alternative, accumulating evidence suggests that TMBPF may exert multiple toxicological effects, raising concerns about its potential developmental neurotoxicity. The present study aimed to investigate the neurodevelopmental effects of TMBPF using both in vitro and in vivo approaches. First, a developmental neurotoxicity assay employing Sox1−GFP mouse embryonic stem cells was used to evaluate cytotoxicity using the cell counting kit-8 assay and neural differentiation based on green fluorescent protein (GFP) fluorescence intensity. The results indicated developmental neurotoxic potential according to the established discrimination index. Subsequently, pregnant and lactating mice were exposed to TMBPF daily from gestational day 10.5 to postnatal day 20, and their offspring were assessed for behavioral performance as well as changes in the expression of neurodevelopment-related genes in the brain. Behavioral analyses encompassed multiple domains, including memory and learning, social behavior, anxiety-related responses, and spontaneous locomotor activity, suggesting alterations in these functional outcomes. Molecular analyses further demonstrated changes associated with dopaminergic and cholinergic signaling, synaptic plasticity, neuronal activity markers, neuropeptides, and inflammatory pathways. Collectively, these findings provide the first evidence in a mammalian model that maternal exposure to TMBPF may influence offspring neurodevelopment. These findings suggest potential implications for human exposure to TMBPF, particularly through food-contact materials, and warrant further mechanistic and dose–response studies.

## 1. Introduction

Bisphenol A (BPA) has been widely used for decades in various industrial applications, including plastics, coatings, printed circuit boards, and thermal papers, owing to its economic advantages, chemical stability, and excellent processability [1]. However, accumulating evidence has demonstrated that BPA acts as an endocrine-disrupting chemical (EDC), exerting hormone-like activities and causing a wide spectrum of toxicities, including reproductive, metabolic, immune, cardiovascular, developmental neurotoxic, and carcinogenic effects [2]. Consequently, BPA is now recognized as a representative EDC; its use has been increasingly restricted, and numerous alternatives have been developed [3]. These substitutes include non-bisphenol polymers such as polyethersulfone (PES), which is derived from bisphenol S-based materials, and polypropylene (PP), a bisphenol-free polymer, as well as structural analogues such as bisphenol F (BPF), bisphenol S (BPS), and bisphenol AF (BPAF) [4,5,6]. Although marketed as “BPA-free,” many of these compounds have since been shown to possess toxic effects, in some cases exceeding those of BPA [7].

Tetramethyl bisphenol F (TMBPF) is one of the major BPA substitutes and is a BPF derivative with four methyl substitutions [8]. Due to its structural and chemical similarity to BPA, it has been widely applied as a key component of epoxy resins, particularly in food-can coatings, and has also found applications in electronic circuit boards, industrial adhesives, and heat-resistant polymers. Early studies suggested that TMBPF exhibited minimal estrogenic activity and demonstrated lower migration levels into food compared with BPA, contributing to its reputation as a safer alternative [9]. However, recent toxicological studies have demonstrated multiple adverse effects of TMBPF, including cytotoxicity, mitochondrial dysfunction, and endocrine-disrupting activity, raising concerns regarding its safety [8,10,11].

TMBPF has been shown to exhibit markedly higher cytotoxicity than BPA, induce apoptosis, disrupt mitochondrial function, and accelerate cellular aging in mammalian cells and *Caenorhabditis elegans* models [10,12]. Additional studies have demonstrated that TMBPF exerts estrogenic and androgenic activity, interferes with thyroid and growth hormone pathways, impairs skeletal homeostasis, functions as a potential obesogen, induces malformations and neurotoxicity, and alters gut microbiota and host metabolism [8,11,13,14,15,16,17,18,19]. These findings suggest that TMBPF may induce toxic effects comparable to, or potentially greater than, those of BPA, raising concerns regarding its safety as a BPA alternative [10].

Currently, no universally established regulatory threshold (e.g., tolerable daily intake or reference dose) for parent TMBPF has been reported by major regulatory agencies such as the European Food Safety Authority (EFSA) or the United States Environmental Protection Agency (EPA). However, toxicological reference values are available. A 90-day repeated-dose oral toxicity study reported no-observed-adverse-effect levels (NOAELs) of 750 mg/kg-bw/day in female rats and 1000 mg/kg-bw/day in male rats, providing a reference point for interpreting currently reported exposure levels and their potential health significance [20].

Growing evidence has also linked long-term EDC exposure to increased prevalence of neurodevelopmental disorders, including autism spectrum disorder (ASD) and attention-deficit/hyperactivity disorder (ADHD) [21,22]. Bisphenols used in plastics, coatings, and packaging materials can migrate into food and beverages, resulting in continuous dietary exposure, with infants and children being particularly vulnerable [23]. Feeding bottles, for instance, may release increased levels of bisphenols when warm liquids are stored or heated [24]. Maternal exposure during gestation and lactation can further transfer these chemicals to offspring, raising concerns about developmental neurotoxicity [25]. Although BPA-free products are widely adopted, mounting evidence indicates that many BPA substitutes may also induce neurodevelopmental toxicity [26].

Experimental models have increasingly suggested that TMBPF may exert neurodevelopmental toxicity. In zebrafish embryos, TMBPF exposure inhibited neurogenesis, impaired dopaminergic neuronal development, and altered locomotor behavior [11]. In chick embryos, even low concentrations caused craniofacial malformations and growth retardation, while in *C. elegans*, accelerated neuronal aging and mitochondrial dysfunction were observed [12,18]. In human stem cell models, low concentrations of TMBPF induced significant apoptosis [10]. These findings suggest that TMBPF, despite being used as a BPA substitute, may pose significant risks to neural development, underscoring the urgent need for rigorous toxicological evaluation.

Given the widespread use of TMBPF and its potential developmental neurotoxicity, comprehensive assessment is required to clarify its possible links to neurodevelopmental disorders. However, evidence regarding TMBPF-induced neurodevelopmental toxicity remains limited, with no systematic mammalian studies conducted to date. Maternal exposure during gestation and lactation represents a critical window within the framework of the developmental origins of health and disease (DOHaD), highlighting the importance of evaluating early-life exposure to TMBPF and its potential long-term neurodevelopmental consequences [27]. This study represents the first integrated in vitro–in vivo evaluation of TMBPF-induced developmental neurotoxicity in a mammalian system. Therefore, the present study aimed to investigate the potential effects of TMBPF on neurodevelopment by integrating in vitro and in vivo approaches. A developmental neurotoxicity (DNT) assay using Sox1−GFP embryonic stem cells was employed to screen for toxicity, followed by assessment of behavioral outcomes and brain-specific gene expression profiles in offspring exposed maternally during gestation and lactation. Through this integrated approach, the study sought to characterize behavioral and molecular alterations associated with TMBPF exposure in a mammalian model.

## 2. Results

### 2.1. Developmental Neurotoxicity Assessment of TMBPF Using Sox1-GFP Cells

**Figure 1:**
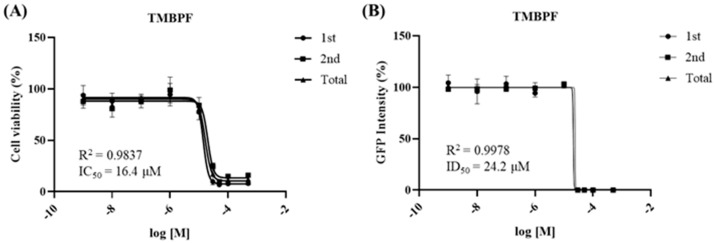
Developmental neurotoxicity test of TMBPF using Sox1−GFP cells. (**A**) To evaluate the neurotoxicity of TMBPF, the IC_50_ value (16.4 μM) was determined using a cytotoxicity test based on the CCK−8 assay. (**B**) The ID_50_ value (24.2 μM) was obtained from a differentiation toxicity test based on GFP intensity measurement. Both assays showed a clear dose-dependent decrease, and curves were fitted using nonlinear regression. Each concentration was tested in six replicates (*n* = 6), and data are expressed as mean ± SEM. According to the discrimination equation, TMBPF was determined to exhibit neurotoxicity.

Before conducting in vivo animal experiments, the developmental neurotoxicity of TMBPF was evaluated using a Sox1−GFP−based in vitro assay. This assay evaluates cytotoxicity and differentiation toxicity, with cell viability measured using the Cell Counting Kit-8 (CCK−8) assay and neural differentiation assessed based on green fluorescent protein (GFP) fluorescence intensity. From these assays, the half-maximal inhibitory concentration (IC_50_) and half-maximal differentiation inhibitory concentration (ID_50_) values are calculated and applied to a discrimination equation to determine developmental neurotoxicity. The IC_50_ value obtained from the cytotoxicity assay was 16.4 μM, while the ID_50_ value determined from the differentiation assay was 24.2 μM (Figure 1). When these values were applied to the revised discrimination equation during the secondary validation step, the calculated score was −1.380129, which is below zero, indicating that TMBPF exhibits developmental neurotoxicity.

### 2.2. Effects of Maternal TMBPF Exposure on General Health and Brain Development

**Figure 2:**
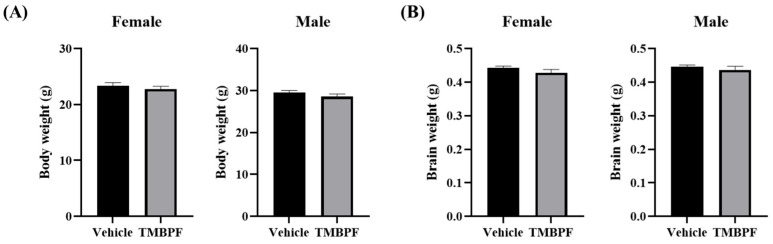
Body weight and brain weight at the final day of the experiment. To evaluate growth and general condition, (**A**) body weight and (**B**) brain weight of the TMBPF-exposed group were compared with those of the vehicle group (*n* = vehicle—female 14, male 14, TMBPF—female 14, male 9). Data were analyzed per individual animal (not litter-based). Data are presented as mean ± SEM and analyzed using a Student’s *t*-test.

Throughout the study period following group separation, no abnormal behaviors were observed in the animals. At the end of the experimental period, no statistically significant differences in body weight were detected between the vehicle and TMBPF-treated groups (Figure 2A). In addition, necropsy revealed no remarkable gross pathological findings in any organs. Brain weights measured during tissue preparation also showed no significant differences between the two groups (Figure 2B). These findings indicate that TMBPF exposure did not affect general somatic growth or brain development under the present conditions.

### 2.3. Effects of Maternal TMBPF Exposure on Memory and Learning Abilities

**Figure 3:**
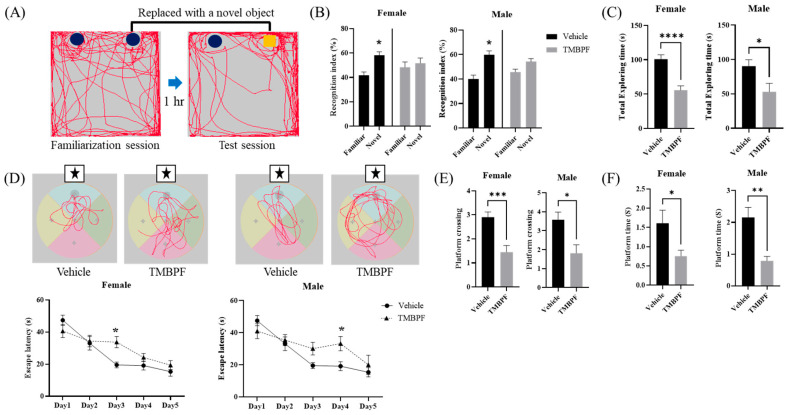
Behavioral assessments of memory and learning abilities following TMBPF exposure. The novel object recognition test (**A**–**C**) and Morris water maze test (**D**–**F**) were conducted to evaluate the effects of TMBPF on cognitive function. (**A**) The familiarization and test sessions were performed one hour apart to assess recognition of familiar versus novel objects. (**B**) Both male and female mice in the TMBPF group showed significantly reduced preference for the novel object and (**C**) shorter total exploration times compared to the vehicle group, although the degree of reduction varied between sexes. (**D**) In the Morris water maze test, mice underwent five days of training followed by a probe test on day 6 to evaluate memory retention for the platform location. (**E**) The number of platform crossings and (**F**) the time spent in the platform area in vehicle and TMBPF-exposed mice. Symbols indicate specific positions: circles represent object locations, squares indicate the novel object, and stars indicate the target zone or platform area. Data are expressed as mean ± SEM. Sample sizes varied by test: novel object recognition, *n* = vehicle: female 14, male 14; TMBPF: female 13, male 9; Morris water maze, *n* = vehicle: female 14, male 14; TMBPF: female 14, male 9. Data were analyzed per individual animal. Statistical analyses were performed using GraphPad Prism 10 software. Behavioral data were analyzed using Student’s unpaired t-test, except for Morris water maze training (trial) data, which were analyzed by two-way ANOVA followed by Bonferroni’s post hoc test (* *p* < 0.05, ** *p* < 0.01, *** *p* < 0.001, and **** *p* < 0.0001 vs. vehicle).

Memory function was evaluated using the novel object recognition test (Figure 3A–C). The recognition index, defined as the proportion of time spent exploring the novel object relative to the familiar one, was reduced in both male and female TMBPF-treated groups, showing no significant preference for the novel object. This pattern may reflect altered novelty recognition or object discrimination performance. In addition, total exploration time was significantly decreased in TMBPF-treated males and females compared with the vehicle group, suggesting reduced exploratory behavior and potential changes in cognitive-related performance.

The Morris water maze was conducted to assess memory and learning ability (Figure 3D–F). Mice were trained for five consecutive days from different starting quadrants, excluding the target quadrant containing the hidden platform. On day 6, the platform was removed, and spatial memory performance was evaluated by platform crossings and time spent in the target quadrant. During the training period, escape latency was significantly increased in females on day 3 and in males on day 4 compared with the vehicle group. In the probe trial on day 6, both male and female TMBPF-treated mice showed significant reductions in platform crossings and time spent in the target quadrant, indicating altered spatial memory-related performance. Collectively, these findings suggest that maternal TMBPF exposure may influence memory, learning, and cognitive-associated behaviors in offspring.

### 2.4. Alterations in Social Behavior Following Maternal TMBPF Exposure

**Figure 4:**
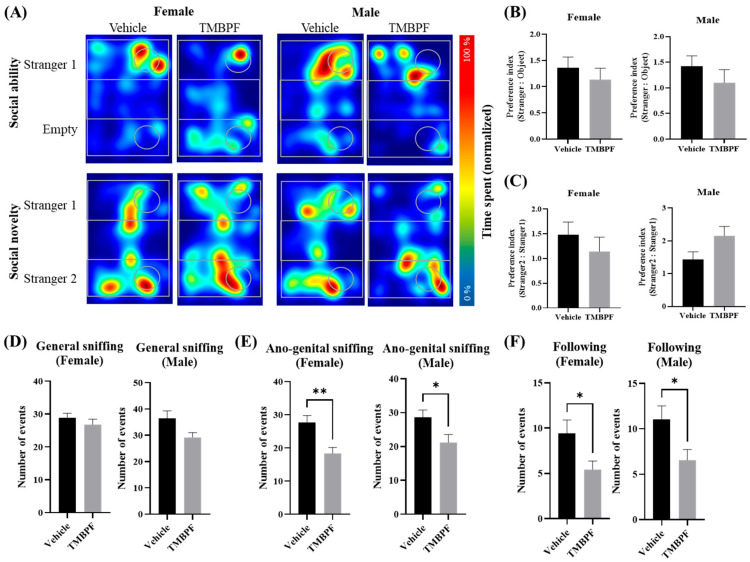
Assessment of social behavior in TMBPF-exposed offspring. To assess the effects of TMBPF on social behavior, the three-chamber test (**A**–**C**) and the direct social interaction test (**D**–**F**) were conducted. (**A**) The three-chamber test consisted of two phases: the sociability phase and the social novelty phase. (**B**) In the sociability phase, the time spent exploring an empty chamber versus a chamber containing a stranger mouse was measured. (**C**) In the social novelty phase, preference between a familiar mouse (stranger 1) and a novel mouse (stranger 2) was assessed. The social interaction test measured (**D**) general sniffing, (**E**) anogenital sniffing, and (**F**) following behavior. Data are expressed as mean ± SEM. Sample sizes varied by test: three-chamber test, *n* = vehicle: female 14, male 14; TMBPF: female 14, male 8; social interaction test, *n* = vehicle: female 14, male 14; TMBPF: female 14, male 9. Data were analyzed per individual animal. Statistical analyses were performed using GraphPad Prism 10 software with Student’s *t*-test (* *p* < 0.05 and ** *p* < 0.01 vs. vehicle).

Sociability and social novelty were assessed using the three-chamber test (Figure 4A–C). No significant changes in sociability were observed in the TMBPF-treated groups compared with the vehicle group, and no pronounced adverse effects were detected in either sex. The social interaction test evaluated general sniffing, anogenital sniffing, and following behavior toward a stranger mouse (Figure 4D–F). Significant reductions were observed in most parameters in the TMBPF-treated groups, with the exception of general sniffing. Compared with the three-chamber test, these findings suggest altered social interaction-related behaviors following TMBPF exposure. Collectively, these results indicate that maternal TMBPF exposure may influence specific aspects of social behavior in offspring, although the magnitude of changes varied across tests and behavioral parameters.

### 2.5. Effects of TMBPF Exposure on Anxiety-Related Behavior and Locomotor Activity

**Figure 5:**
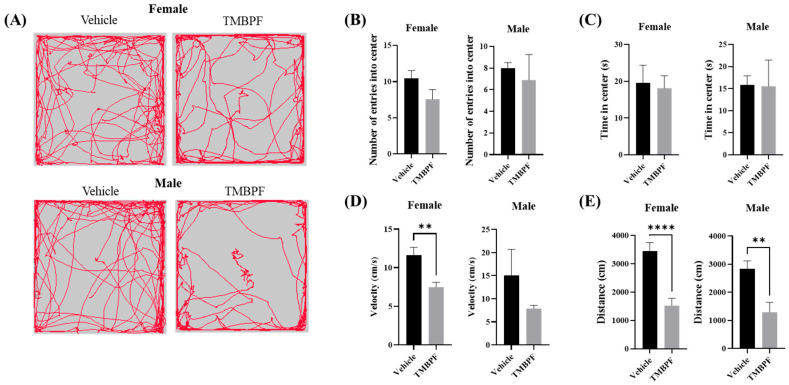
Assessment of anxiety-like effects in TMBPF-exposed offspring. The open field test was conducted to assess whether TMBPF exposure affects anxiety and spontaneous locomotion. (**A**) The test was performed in a square box measuring 60 × 60 cm for 5 min, (**B**) during which mouse movements were tracked to measure the number of entries into the central area (30 × 30 cm), (**C**) the time spent in the center, (**D**) movement velocity, and (**E**) total distance traveled. Data are presented as mean ± SEM (*n* = vehicle: female 14, male 14; TMBPF: female 14, male 9), and statistical analyses were conducted per individual animal. Statistical analyses were performed using GraphPad Prism 10 software with Student’s *t*-test (** *p* < 0.01, and **** *p* < 0.0001 vs. vehicle).

Anxiety-related behavior and spontaneous locomotion were evaluated using the open field test by measuring center entries, time spent in the center, movement speed, and total distance traveled (Figure 5). No statistically significant differences were observed between the vehicle and TMBPF-treated groups in center entries or time spent in the center. However, movement speed was significantly reduced in females, and total distance traveled was significantly reduced in both sexes. These results suggest that TMBPF exposure was not associated with detectable changes in anxiety-related parameters under the present conditions, but was associated with reduced locomotor activity in male and female mice.

### 2.6. Effects of TMBPF Exposure on Depression-like Behavior, and Cognitive Function

**Figure 6:**
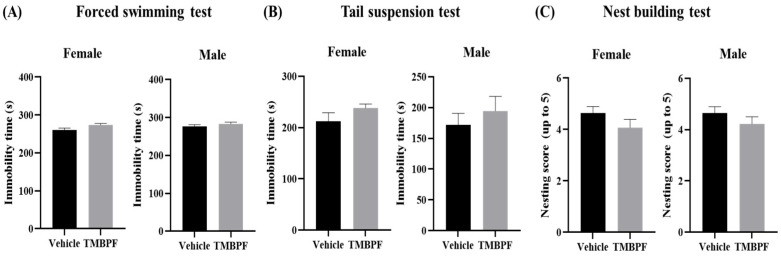
Assessment of depression-like, helplessness, and voluntary behaviors in TMBPF-exposed offspring. To examine whether TMBPF induces depression and helplessness, (**A**) the forced swimming test and (**B**) tail suspension test were conducted, and (**C**) the nest building test was performed to assess cognitive function and voluntary behavior. Data are presented as mean ± SEM (*n* = vehicle: female 14, male 14; TMBPF: female 14, male 9), and statistical analyses were conducted per individual animal. Statistical analyses were performed using GraphPad Prism 10 software with Student’s *t*-test.

Depression- and helplessness-like behaviors were assessed using the forced swimming test and tail suspension test by measuring immobility time under stress conditions (Figure 6A,B). Both tests evaluate depressive- and helplessness-like behaviors by measuring the degree to which an animal ceases attempts to escape when placed in an inescapable stressful situation. In both tests, the TMBPF-treated groups showed an increasing trend in immobility time compared with the vehicle group in females and males. Although the tendency appeared more pronounced in the tail suspension test than in the forced swimming test, the differences did not reach statistical significance. These findings suggest that TMBPF exposure was not associated with statistically detectable alterations in depression- or helplessness-like behavior under the present conditions.

In the nest-building test, both male and female TMBPF-treated groups showed slightly lower scores than the vehicle group based on the 1–5 rating scale evaluating nest structure quality and engagement in nest-building behavior; however, these differences were not statistically significant (Figure 6C). Together, these results indicate that TMBPF exposure did not produce statistically significant changes in this measure of nest-building performance.

### 2.7. Effects of Maternal TMBPF Exposure on Gene Expression in the Brain

#### 2.7.1. Neurodevelopmental and Neurobehavioral Associated Gene Expression

**Figure 7:**
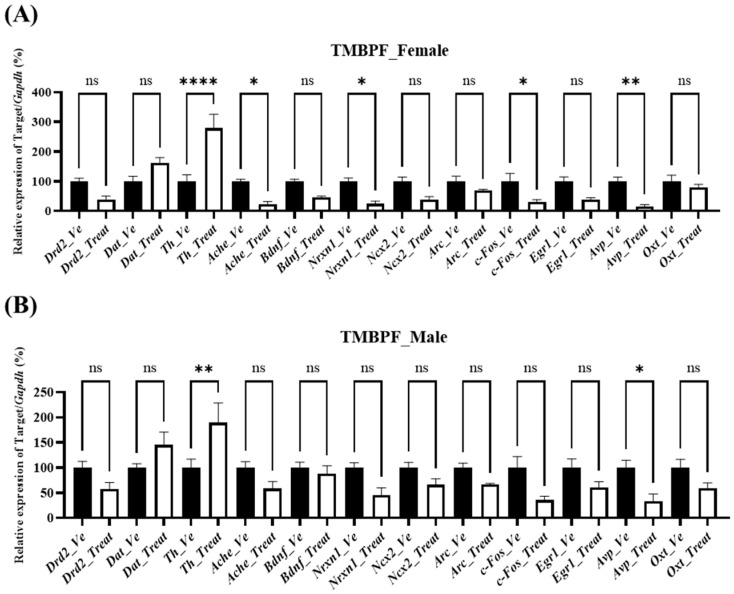
Sex-specific changes in neurodevelopmental and neurobehavioral gene expression after TMBPF exposure. Changes in the expression of neurodevelopmental and neurobehavioral genes following TMBPF exposure were examined (**A**) in female brain samples and (**B**) male brain samples. The genes are grouped as follows: neurotransmission-related (*Drd2*, *Dat*, *Th*, *Ache*), synaptic plasticity and neurodevelopment (*Bdnf*, *Nrxn1*, *Ncx2*, *Arc*), neuronal activity markers (*c-Fos* and *Egr1*), and neuropeptides (*Avp* and *Oxt*). Data are presented as mean ± SEM (*n* = Vehicle—Female 10, Male 10, TMBPF—Female 10, Male 6), and statistical analyses were conducted per individual animal. Statistical analysis was performed using one-way ANOVA followed by Bonferroni’s post hoc test. in GraphPad Prism 10 (ns, not significant; * *p* < 0.05, ** *p* < 0.01, and **** *p* < 0.0001 vs. vehicle).

In females, expression changes were observed across multiple genes (Figure 7). Among neurotransmission-related genes, acetylcholinesterase (*Ache*) was significantly decreased, whereas tyrosine hydroxylase (*Th*) was significantly increased. Among neuronal activity markers, fos proto-oncogene (*c-Fos*) was significantly decreased. Within neuropeptides, arginine vasopressin (*Avp*) was significantly reduced, whereas oxytocin (*Oxt*) remained unchanged. In males, *Th* was significantly increased, and *Avp* was significantly decreased. In addition, *Ache*, neurexin 1 (*Nrxn1*), and *c-Fos* exhibited decreasing trends, although these changes did not reach statistical significance. Overall, a gene expression pattern comparable to that observed in females was identified.

#### 2.7.2. Inflammation-Associated Gene Expression

**Figure 8:**
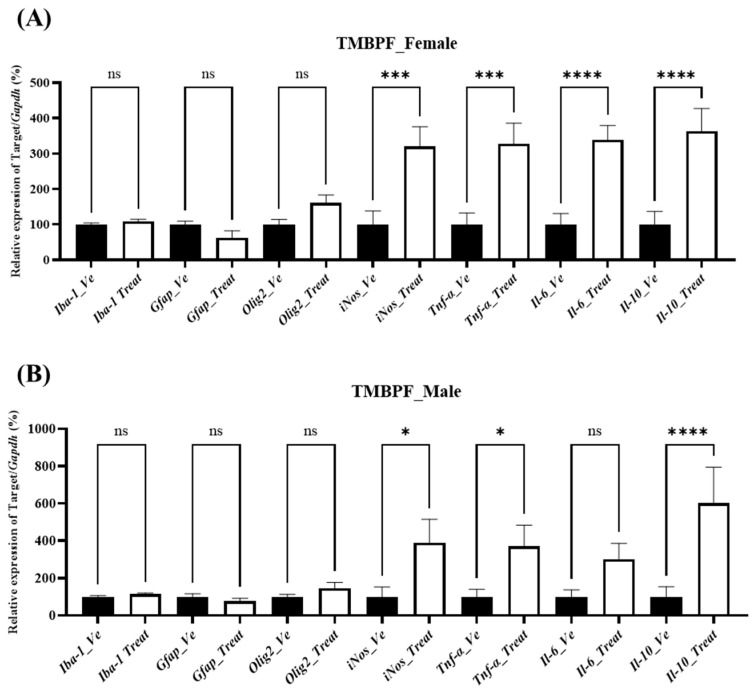
Analysis of brain inflammation-related gene expression following TMBPF exposure. The expression levels of *Iba-1* and *Gfap*, which are activation markers for astrocytes and microglial cells, respectively, as well as inflammation-related cytokines (*Tnf-α*, *Il-6*, *Il-10*), *iNos*, and the oligodendrocyte transcription factor *Olig2* were examined in brains of (**A**) female and (**B**) male mice. Data are presented as mean ± SEM (*n* = Vehicle—Female 10, Male 10, TMBPF—Female 10, Male 6), and statistical analyses were conducted per individual animal. Statistical analysis was performed using one-way ANOVA followed by Bonferroni’s post hoc test in GraphPad Prism 10 (* *p* < 0.05, *** *p* < 0.001 and **** *p* < 0.0001 vs. vehicle).

To assess whether neuroinflammatory processes were associated with the observed behavioral and neurodevelopmental changes, the expression levels of inflammation-related genes were examined (Figure 8). No statistically significant changes were detected in glial cell markers, including ionized calcium-binding adapter molecule 1 (*Iba1*; microglial marker), glial fibrillary acidic protein (*Gfap*; astrocyte marker), and oligodendrocyte transcription factor 2 (*Olig2*; oligodendrocyte marker), in either sex following TMBPF exposure. In contrast, transcriptional changes were observed in several inflammatory and oxidative stress–related genes. The expression of inducible nitric oxide synthase (*iNos*) and the cytokines tumor necrosis factor alpha (*Tnf-α*) and interleukin 10 (*Il-10*) was significantly increased in both females and males, while interleukin 6 (*Il-6*) was significantly elevated in females compared with the vehicle group. To confirm the corresponding protein-level changes, ELISAs were performed for TNF-α and IL-6; however, their concentrations were below the detection limit.

### 2.8. Integrated Summary of Behavioral and Molecular Alterations

To facilitate interpretation of the results, an integrated summary of the behavioral and molecular findings is provided. TMBPF exposure was associated with changes in memory and learning, alterations in social interaction, and reduced spontaneous locomotor activity. These behavioral changes were accompanied by alterations in key neurodevelopment-related pathways. In particular, dopaminergic signaling appeared to be altered, as indicated by changes in the expression of Th and Ache, which may be associated with reduced locomotor activity and motivational processes. In addition, alterations in synaptic plasticity-related genes such as *Nrxn1* may be related to changes in cognitive and social functions. Changes in neuropeptides, including *Avp*, may also contribute to alterations in social behavior and emotional regulation. Although markers of oxidative stress and inflammation were elevated, the lack of corresponding protein-level changes indicates that these pathways may have a limited or secondary contribution to the observed behavioral outcomes.

## 3. Discussion

In the present study, maternal exposure to TMBPF resulted in neurobehavioral alterations in offspring, including impairments in memory and learning, changes in social interaction, and reduced spontaneous locomotor activity. These behavioral changes were accompanied by alterations in the expression of genes related to neurotransmission, neuronal activity, and neuropeptides. Notably, these effects were observed in the absence of significant changes in body or brain weight, indicating that TMBPF exposure did not affect general somatic growth or brain development under the present conditions.

Before conducting the animal study, a DNT assay using Sox1−GFP cells was performed to initially evaluate neurodevelopmental toxicity at the cellular level. Sox1 is a critical gene in early neural differentiation, and reduced expression reflects impairment in neurodevelopment [28]. The Sox1−GFP–based assay is an established alternative test method for developmental neurotoxicity, with reported high reproducibility and predictive performance [29]. This system has been validated and applied in the assessment of various endocrine-disrupting chemicals, including BPA [30]. GFP tagging enables quantitative measurement of differentiation inhibition, which, together with cytotoxicity data and calculation of a discrimination index, provides a reliable platform for identifying developmental neurotoxicity [29,30]. In the present study, TMBPF induced both cytotoxicity and inhibition of differentiation, and the resulting negative discrimination index supported neurodevelopmental toxicity. Notably, TMBPF exhibited lower IC_50_ and ID_50_ values than previously reported for BPA, suggesting comparatively stronger toxicity [29].

Subsequently, TMBPF was administered to dams during gestation and lactation, and behavioral and neurological changes were evaluated in the offspring. Behavioral assessments began approximately three weeks after weaning. Memory and learning abilities, assessed by the novel object recognition and Morris water maze tests, showed a decreasing tendency in both sexes, with more pronounced effects observed in females (Figure 3). Regarding social behavior, no significant differences were detected in either sex in the three-chamber test; however, the social interaction test revealed alterations in two parameters in both sexes, without marked sex-specific differences (Figure 4).

Notably, the open field test showed no significant changes in anxiety-related behavior in either sex, whereas spontaneous locomotor activity was reduced in both males and females, with a more pronounced decrease observed in females (Figure 5). In contrast, depressive-like behavior, helplessness, and voluntary behavior were not significantly affected in either sex (Figure 6). These findings are consistent with previous zebrafish studies reporting a reduction in locomotor activity following TMBPF exposure. In those studies, oxidative stress, impaired neurodevelopment, and alterations in dopaminergic gene expression were proposed as underlying mechanisms, suggesting that similar mechanisms may contribute to the behavioral abnormalities observed in the present study [11]. These results suggest that the observed reduction in locomotor activity may reflect alterations in motivation- or neurotransmission-related processes rather than general motor impairment. In particular, changes in dopaminergic signaling, as indicated by gene expression alterations observed in this study, may underlie the behavioral effects and contribute to the more pronounced responses observed in females.

Gene expression changes associated with behavioral alterations were examined in the brain (Figure 7). Given the limited available evidence on TMBPF-induced neurodevelopmental effects, a broad range of pathways was explored to identify potential targets. For neurotransmission-related pathways, the expression of key dopaminergic and cholinergic genes, including dopamine receptor D2 (*Drd2*), dopamine transporter (*Dat*), *Th*, and *Ache*, was analyzed, as these genes collectively regulate dopamine signaling, synthesis, reuptake, and acetylcholine degradation [31,32,33]. In female offspring exposed to TMBPF, alterations in gene expression were detected, although statistically significant changes were limited to a decrease in *Ache* and an increase in *Th*. In males, only *Th* showed a significant increase, although other genes exhibited similar trends. The observed increase in *Th* may represent a compensatory response. *Ache* expression was significantly decreased in females and showed a decreasing trend in males; given the role of acetylcholine in learning, memory, arousal, and attention, this reduction may reflect a compensatory interaction with dopaminergic signaling [34]. Given the roles of dopamine and acetylcholine in motor control, learning, memory, arousal, and attention, these changes may indicate disruption of dopaminergic and cholinergic neurotransmission, which could contribute to the reduced spontaneous locomotor activity and impairments in learning and memory observed in the offspring. Notably, a previous zebrafish study of TMBPF-induced neurodevelopmental toxicity also reported increased *Th* expression, further supporting the relevance of this finding for future investigations.

For genes associated with synaptic plasticity and neurodevelopment, the expression of brain-derived neurotrophic factor (*Bdnf*), *Nrxn1*, sodium calcium exchanger 2 (*Ncx2*), and activity-regulated cytoskeleton-associated protein (*Arc*) was analyzed (Figure 7). *Bdnf* is an essential neurotrophin for neuronal survival and synaptic strengthening, *Nrxn1* plays a key role in synaptic structure and pre- and postsynaptic adhesion, and *Ncx2* regulates intracellular calcium levels and contributes to synaptic stability as well as neuronal differentiation and migration during development [35,36,37]. In the present study, decreasing trends were observed across multiple genes in females, but statistical significance was reached only for *Nrxn1*. In males, no genes showed statistically significant alterations, although *Nrxn1* exhibited a decreasing tendency. These patterns may indicate potential disturbances in synaptic plasticity and connectivity, and the reduction in *Nrxn1* expression may provide a molecular basis for deficits in learning, memory, and social cognition.

With respect to neuronal activity, the expression of *c-Fos* and early growth response 1 (*Egr1*) was examined (Figure 7). *c-Fos* is an immediate early gene rapidly induced by neuronal stimulation, while *Egr1* is a transcription factor involved in synaptic long-term potentiation and memory formation [38]. In this study, female offspring exposed to TMBPF exhibited decreasing trends in both *c-Fos* and *Egr1* expression, although statistical significance was reached only for *c-Fos*. In males, no statistically significant changes were detected, while a similar decreasing tendency was observed. These findings indicate that TMBPF exposure may be associated with altered neuronal activity, and the reduction in *c-Fos* expression may reflect impaired neuronal responsiveness, potentially affecting activity-dependent synaptic plasticity and information processing.

Among neuropeptides, the expression of *Avp* and *Oxt* was examined (Figure 7). Peripherally, *Avp* functions as an antidiuretic hormone that promotes renal water reabsorption, whereas in the central nervous system it regulates social behavior, stress responses, and aggression [39]. *Oxt*, in turn, is known peripherally for its role in uterine contraction and lactation, but centrally it acts as a neuropeptide that modulates social bonding and emotional stability [40]. Reductions in these neuropeptides are therefore associated with heightened anxiety and impaired sociability. In the present study, *Avp* expression was decreased in both male and female offspring exposed to TMBPF. These findings suggest that TMBPF exposure may influence neuropeptide systems critical for regulating social behavior and emotional control, and the decrease in *Avp* expression may be associated with altered social interaction and emotional regulation, consistent with the behavioral abnormalities observed.

TMBPF, as an EDC, may involve oxidative stress, inflammation, or hormonal signaling interference that acts in concert with, or contributes to, the observed changes in the brain (Figure 8). In this study, the expression of oxidative stress markers, including *iNos*, as well as pro-inflammatory cytokines such as *Tnf-α* and *Il-6*, together with the anti-inflammatory cytokine *Il-10*, was examined [41]. In addition, markers of immune activity specific to brain-resident cells, including microglia (*Iba-1*), astrocytes (*Gfap*), and oligodendrocytes (*Olig2*), were evaluated [42]. Among these, *iNos*, *Tnf-α*, and *Il-10* were significantly upregulated in both sexes, and *Il-6* was elevated in females, whereas *Iba-1*, *Gfap*, and *Olig2* showed no significant changes. However, protein expression of the increased iNos and cytokines was assessed by enzyme-linked immunosorbent assay (ELISA) but found to be below the detection limit; therefore, it is difficult to conclude that neuroinflammation directly accounted for the observed behavioral abnormalities.

From a DOHaD perspective, even subtle alterations in inflammatory or oxidative stress–related pathways during critical developmental windows may contribute to long-term physiological and functional changes [43]. Early-life inflammatory conditions have been shown to induce persistent effects on organ development and disease susceptibility later in life. Although overt neuroinflammation was not evident in the present study, the observed changes in inflammatory markers may reflect early-life programming events that increase vulnerability to subsequent neurological dysfunction. Taken together, these findings suggest that severe neuroinflammation was unlikely to be present. However, oxidative stress may have contributed to the activation of immune-related pathways, potentially increasing vulnerability of neural systems. Future studies using validated neuroinflammation models (e.g., lipopolysaccharide (LPS)-induced paradigms) may help clarify the contribution of inflammatory processes to the observed effects.

The prevalence of neurodevelopmental and behavioral disorders has been increasing steadily [44]. From a physiological and toxicological perspective, dietary exposure to endocrine-disrupting chemicals derived from food packaging materials has been proposed as one of several potential contributing factors [45]. TMBPF is widely used as a BPA substitute, particularly in food-can coatings, making it relevant to potential human exposure through the oral route [18]. Although TMBPF was initially introduced as a safer alternative, an increasing number of toxicological studies have reported biological effects associated with its exposure. While the causal relationship between TMBPF and neurodevelopmental disorders remains to be fully established, accumulating evidence, including the present findings, suggests that further investigation into its neurodevelopmental safety profile is warranted.

The results of this study suggest that maternal exposure to TMBPF may influence neurodevelopmental and behavioral outcomes in offspring, including alterations in memory, learning ability, sociability, and spontaneous locomotion. These behavioral patterns were accompanied by changes in the expression of genes related to neurotransmission, synaptic plasticity, neuronal activity, and neuropeptides. This study has several strengths, including the integrated use of in vitro and in vivo approaches, the combined evaluation of behavioral and molecular endpoints, and the application of a mammalian model to investigate the neurodevelopmental effects of TMBPF. To our knowledge, this study provides the first evidence from a mammalian model supporting the potential neurodevelopmental effects of TMBPF previously indicated by alternative test systems and in vitro approaches. Nevertheless, as this study was designed as an exploratory prescreening investigation, several limitations should be considered. Due to the limited scale of the study, data were compared using individual offspring rather than dams/litters. In addition, only a single dose level was evaluated, and molecular analyses were performed using whole-brain samples. Given the functional specificity of distinct brain regions, further studies should focus on region-specific mechanisms, combining gene and protein expression analyses with spatial distribution within brain tissues. From a translational perspective, considering the widespread use of TMBPF in food-contact materials such as can coatings, further investigation is needed to better characterize human exposure scenarios and potential exposure levels. Establishing dose–response relationships, evaluating region-specific effects in the brain, and validating findings at the protein level will be essential to bridge the gap between experimental findings and human health risk assessment. Such approaches will provide a clearer and more in-depth understanding of the mechanisms underlying TMBPF-induced neurodevelopmental toxicity, and the present study offers an important foundation for future work.

## 4. Materials and Methods

### 4.1. Chemical

TMBPF (Cat. No. M1099) was purchased from Tokyo Chemical Industry Co., Ltd. (Tokyo, Japan).

### 4.2. Mouse Embryonic Stem Cells (mESCs) Culture and Neuronal Differentiation

Sox1−GFP mESCs kindly obtained from Prof. Eekhoon Jho (Cellular Signalling Transduction Laboratory, University of Seoul) were expanded on gelatinised (0.2%) 100 mm Petri dishes (Corning Life Sciences, Corning, NY, USA) at 37 °C, 5% CO_2_. Basal culture medium was Dulbecco’s Modified Eagle Medium (DMEM; Welgene, Gyeongsan, Korea), supplemented with 15% heat-inactivated fetal bovine serum (FBS; Biowest, Nuaillé, France), 2 mM L-glutamine, 0.1 mM β-mercaptoethanol (Sigma-Aldrich, St. Louis, MO, USA), non-essential amino acids (NEAA; 1×), penicillin (100 U mL^−1^)/streptomycin (100 µg mL^−1^) and 1000 U mL^−1^ mouse leukemia inhibitory factor (mLIF; Miltenyi Biotec, Bergisch Gladbach, Germany). Neural differentiation was performed using a free-floating embryoid-body protocol, based on previously described methods [46]. Cells (4 × 10^4^ cells well^−1^) were seeded into ultra-low-attachment 96-well U-bottom plates (Corning, Corning, NY, USA) with serum-free DMEM/F-12 containing 1× N2, 1× B27, 7.5% BSA (fraction V), 2 mM L-glutamine and antibiotics. Half the medium was replaced every other day.

### 4.3. Developmental Neurotoxicity Test

Developmental neurotoxicity (DNT) classification was based on the discriminant function: 0.4345932 × logIC_50_ + 0.4295667 × logID_50_ + 2.687087. Using this model, we evaluated whether the tested chemical induced DNT by determining IC_50_ and ID_50_ values through a cell viability test and neurodifferentiation analysis. Depending on the solubility test, the TMBPF was dissolved in ethanol as a vehicle to prepare a 100 mM stock solution for use in the experiments. The final concentration of the vehicle was maintained below 0.5%, a level known to be non-toxic. TMBPF was tested at concentrations of 0.001, 0.01, 0.1, 1, 10, 30, 50, 100, and 500 μM. The concentration range was selected to cover a broad range on a logarithmic scale. Based on preliminary experiments, additional intermediate concentrations (30 and 50 μM) were included to more precisely capture the expected IC_50_ range between 10 and 100 μM. A higher concentration (500 μM) was further included to ensure coverage of conditions inducing maximal effects on neurosphere formation.

#### 4.3.1. Cell Viability Assay

Cells (7.5 × 10^3^ cells well^−1^ in 70 µL) were plated in clear 96-well flat-bottom plates (SPL Life Sciences, Gyeonggi-do, Korea). Twenty-four hours later, medium was replaced with 200 µL growth medium containing serial dilutions of TMBPF. After 48 h exposure, DPBS washes were followed by 60 min incubation with CCK-8. Optical density at 450 nm was measured on a Synergy H1 reader (BioTek Instruments, Winooski, VT, USA). Cell viability (%) was calculated versus vehicle controls, and IC_50_ values were determined using GraphPad Prism 10 (GraphPad Software, San Diego, CA, USA).

#### 4.3.2. Neural-Lineage Differentiation Assay

Suspensions of 100 cells were cultured in 140 µL differentiation medium with/without TMBPF in U-bottom, low-attachment 96-well plates (Corning, Corning, NY, USA). On day 4, GFP-positive neurospheres were captured using a Lionheart FX imaging system (BioTek Instruments, Winooski, VT, USA) and analyzed using Gen5 software (BioTek Instruments, Winooski, VT, USA). Relative GFP intensity furnished the ID_50_ value by non-linear curve-fit in GraphPad Prism 10 (GraphPad Software, San Diego, CA, USA).

### 4.4. Experimental Animals

The use of animals in this study was reviewed and approved by the Institutional Animal Care and Use Committee of Chungbuk National University (CBNUA-24-0032-02; 15 July 2024). Ten-week-old specific-pathogen-free C57BL/6N mice (males: 25 ± 2 g; females: 20 ± 2 g) were obtained from Samtako Bio Korea (Osan, Republic of Korea). Mice were housed under controlled conditions with a temperature of 23 ± 2 °C, relative humidity of 50 ± 10%, and a 12-h light/dark cycle (lights on from 8:00 to 20:00). All animals were provided with an AIN-76A diet (Research Diets, New Brunswick, NJ, USA, D10001) and sterilized water ad libitum. After acclimatization, female and male mice were co-housed overnight at a 2:1 ratio for mating. The presence of a vaginal plug was taken as embryonic day (E) 0.5, and the day of birth was defined as postnatal day (PND) 0. All animals were monitored daily throughout the study. Humane endpoints were defined a priori as the appearance of abnormal mobility, signs of pain or distress, or excessive stress-related behaviors such as self-injury. No animals met these criteria during the experimental period.

### 4.5. Dose Selection and Chemical Treatment

The dosage for TMBPF was determined by equimolar conversion from the BPA NOAEL (5 mg/kg/day), considering the molecular weight of TMBPF (256.4 g/mol) and BPA (228.3 g/mol). The resulting equivalent dose was 5.6 mg/kg/day. Pregnant mice received daily subcutaneous injections of TMBPF dissolved in corn oil from E10.5 to PND20. Control dams received the same volume of vehicle (corn oil) under identical conditions. The injection volume was adjusted to 10 mL/kg body weight. Offspring were obtained from four vehicle-treated dams and three TMBPF-treated dams. The study groups consisted of vehicle (14 female, 14 male) and TMBPF-treated (14 female, 9 male) offspring. Offspring were assigned to their respective treatment groups at birth according to maternal exposure. Offspring were included in the analysis without additional selection. After weaning, animals were housed according to treatment group and sex, with no more than five animals per cage. A detailed summary of the distribution of offspring by dam, sex, and treatment group is provided in Supplementary Table S1.

### 4.6. Behavioral Analysis

Behavioral testing began at postnatal week 7, when offspring had reached sexual and physiological maturity. To minimize carry-over effects, only one to two tests were conducted per week. For sociability assessments, such as the social interaction test and the three-chamber test, subject mice were introduced to unfamiliar mice of the same sex and age. Table 1 summarizes the behavioral parameters measured, the number of animals used, and the ages at which each assessment was conducted. Detailed procedures for each behavioral test are described in the following subsections. All experiments were performed between 8:00 AM and 4:00 PM in a soundproof room maintained at 25 ± 2 °C, identical to the breeding room conditions. On test days, mice were transferred to the testing room at least 30 min before the start of the experiment for acclimatization. A resting period of at least two days per week was provided to ensure sufficient recovery between tests.

#### 4.6.1. Memory and Learning Abilities

##### Novel Object Recognition Test

To assess object memory, mice were first allowed to explore an empty 60 × 60 × 50 cm acrylic chamber with a white base for 10 min, 24 h before testing. In the familiarization phase, conducted the following day, two identical objects were placed in the arena and the mouse was allowed to explore freely for 5 min. After a 1-h delay, the subject was returned to the arena where one object had been replaced with a novel item. A 5-min testing session followed. Mouse behavior near each object was tracked using EthoVision XT 14 (Noldus Information Technology, Wageningen, The Netherlands), and interaction time was analyzed.

##### Morris Water Maze Test

The Morris water maze task was conducted in a 90 cm diameter, 40 cm deep pool filled with opaque water maintained at 25 ± 2 °C using skim milk powder. The pool was conceptually divided into four quadrants, and the hidden platform was placed in quadrant III, submerged 10 mm beneath the surface and positioned 11 cm from the wall. Training consisted of four trials per day over five days, during which mice were placed into the pool from one of the non-target quadrants (I, II, or IV) in a randomized order. Each trial lasted for a maximum of 60 s. Mice failing to locate the platform within this time were guided to it and allowed a 30-s rest. EthoVision XT 14 software was used to record escape latency and swimming path. On the sixth day, the platform was removed, and a 60-s probe trial was conducted to evaluate spatial memory based on time spent in the target quadrant and platform area crossings.

#### 4.6.2. Social Behavior

##### Three-Chamber Social Test

Three adjoining acrylic compartments (40 cm wide, 20 cm long, 22 cm high) formed the arena, with 10 cm × 5 cm doorways for free movement. After a 5 min habituation with empty wire cups (8 cm × 10 cm) in each side chamber, testing proceeded:

- Sociability phase—a stranger mouse (S1) was secured in one cup; the other remained empty (E). The test mouse explored for 10 min.
- Novelty phase—the empty cup was replaced by a second stranger (S2), and exploration continued for 10 min.

Behavioral videos were processed in EthoVision XT 14 to quantify time near each cup, path length, and zone occupancy. Preference scores were computed as (time near S1—time near E)/(time near S1 + time near E) and, subsequently, (time near S2—time near S1)/(time near S2 + time near S1). Apparatus surfaces were cleaned with 70% ethanol between trials.

##### Social Interaction Test

To assess social behavior, a subject mouse and a same-sex, untreated stranger mouse were introduced simultaneously into a white-floor arena (60 × 60 × 50 cm). The animals were allowed to interact freely for 8 min, and behaviors including general sniffing, anogenital investigation, and following were observed. All tests were recorded for further behavioral quantification.

#### 4.6.3. Anxiety-Related Behavior and Spontaneous Locomotion

##### Open Field Test

To assess general locomotor and anxiety-like behavior, mice were subjected to the open field test in a white plastic chamber (60 cm width × 60 cm length × 50 cm height). At the beginning of each trial, a mouse was placed in the front-center portion of the apparatus facing the wall and allowed to move freely for 5 min. After each session, the field was cleaned with 70% ethanol. Animal trajectories were recorded and quantified using EthoVision XT 14 (Noldus Information Technology, Wageningen, The Netherlands). Time spent in the center zone, center zone entries, total distance, and velocity were analyzed.

#### 4.6.4. Depression-like Behavior and Cognitive Function

##### Forced Swimming Test

Each mouse was tested in a transparent cylinder filled with 1400 mL of water, corresponding to a water level of about 12 cm. The water temperature was controlled at 25 ± 2 °C. Animals were placed gently into the center of the container and observed for 5 min. Immobility duration was analyzed using EthoVision XT 14.

##### Tail Suspension Test

The tail suspension test was conducted by suspending each mouse 50 cm above ground level. Animals were recorded for 6 min, and behavioral scoring focused on the duration of immobility during the last 5 min. Immobility was analyzed using EthoVision XT 14.

##### Nest-Building Test

Each test subject received a single piece of cotton (5 cm square, 2.5 g) in an individual cage and was left for 12 h. Nests were then scored using a standard five-point ordinal scale based on published criteria

- 1 = undisturbed cotton
- 2 = cotton slightly torn but no clear nest structure
- 3 = cotton torn and shallow nest present
- 4 = deeper nest with walls partly constructed
- 5 = well-formed dome with walls and mouse fully enclosed.

Photographs were taken, and scoring was performed by two independent investigators to ensure inter-rater reliability.

### 4.7. Body and Brain Weight Measurement

Body weight was measured immediately prior to sacrifice. Mice were sacrificed one week after completion of the final behavioral assessment, and whole brains were collected and prepared for subsequent analyses. Brain weight was measured immediately after tissue preparation.

### 4.8. RNA Extraction and cDNA Synthesis

Using TRI Reagent (Invitrogen, Thermo Fisher Scientific, Waltham, MA, USA), total RNA was isolated from the brain tissue of mice according to the manufacturer’s instructions. The purity and quantity of RNA were assessed at 260 nm using the Synergy H1 (BioTek Instruments, Winooski, VT, USA). Subsequently, 1 µg of RNA was converted into cDNA with the iScript™ cDNA synthesis kit (Bio-Rad, Hercules, CA, USA).

### 4.9. Quantitative Real-Time Polymerase Chain Reaction (qRT-PCR)

Real-time quantitative PCR analyses were performed on a QuantStudio 3 real-time PCR instrument (Applied Biosystems, Foster City, CA, USA) employing Prime Q SYBR Green master mix supplemented with ROX dye (Genetbio, Daejeon, Republic of Korea). Primer information is summarized in Table 2. For each sample, cycle threshold (Ct) values were obtained automatically, ΔCt values were derived by subtracting the Ct of glyceraldehyde 3-phosphate dehydrogenase (*Gapdh)* from that of the target gene, and relative mRNA levels were computed with the 2^-ΔCt^ formula. Data are presented as fold change relative to *Gapdh*-normalized vehicle controls.

### 4.10. Statistical Analysis

Statistical analyses were performed using GraphPad Prism 10. Behavioral comparisons between vehicle and TMBPF groups were conducted using an unpaired two-tailed Student’s t-test, except for Morris water maze training data, which were analyzed by two-way ANOVA with Bonferroni’s post hoc test. Gene expression data were analyzed using one-way ANOVA followed by Bonferroni’s post hoc test. Because this study was designed as an exploratory and preliminary investigation and the number of available litters per group was limited, statistical analyses were conducted using individual offspring as the experimental unit rather than the litter. Accordingly, a primary outcome measure was not defined a priori. Results are presented as mean ± SEM, and statistical significance was set at *p* < 0.05. Behavioral testing and data analysis were performed by investigators blinded to group allocation. Statistical details on behavioral assays are described in Supplementary Table S2.

## 5. Conclusions

In conclusion, the present study demonstrates that maternal exposure to TMBPF is associated with selective neurodevelopmental and behavioral alterations in offspring. Notably, impairments in memory and learning, reduced sociability, and decreased spontaneous locomotor activity were observed, with some effects appearing more pronounced in females. These behavioral changes were accompanied by alterations in gene expression related to dopaminergic and cholinergic signaling, synaptic plasticity, neuronal activity, and neuropeptide regulation, suggesting that TMBPF exposure may disrupt key neurodevelopmental pathways. Although the present study was designed as an exploratory investigation and does not establish a causal relationship, the findings provide the first integrated evidence from a mammalian model supporting the potential neurodevelopmental effects of TMBPF previously indicated by alternative models. Given the widespread use of TMBPF, particularly in food-contact materials, these results highlight the need for further investigation into its neurodevelopmental safety profile. Future studies should incorporate dose–response analyses, brain region–specific evaluations, and protein-level validation to better elucidate the underlying mechanisms and to support more comprehensive risk assessment.

## Figures and Tables

**Table 1:** Behavioral assessment parameters and testing schedule.

Test	Measurement	Number of Animals per Group	Age (Old)
Open field	Time in center (s)	14 female, 14 male for vehicle 14 female, 9 male for TMBPF	7 weeks
	Number of entries into center (*n*)		
	Distance moved (m)		
	Velocity (cm/s)		
Social interaction	General sniffing (*n*)	14 female, 14 male for vehicle 14 female, 9 male for TMBPF	8 weeks
	Anogenital sniffing (*n*)		
	Following (*n*)		
Tail suspension	Immobile time (s)	14 female, 14 male for vehicle	9 weeks
		14 female, 9 male for TMBPF	
Forced swimming	Immobile time (s)	14 female, 14 male for vehicle	10 weeks
		14 female, 9 male for TMBPF	
Novel object recognition	Time around object (s)	14 female, 14 male for vehicle	11 weeks
		13 female, 9 male for TMBPF	
Nest building	Nesting score	14 female, 14 male for vehicle	12 weeks
		14 female, 9 male for TMBPF	
Three-chamber	Preference index	14 female, 14 male for vehicle	13 weeks
		14 female, 8 male for TMBPF	
Morris water maze	Escape latency (s)	14 female, 14 male for vehicle 14 female, 9 male for TMBPF	14–15 weeks
	Distance moved (m)		
	Velocity (cm/s)		
	Platform crossing (*n*)		
	Platform time (s)		

**Table 2:** Quantitative PCR primer information.

Gene	Sequence (5′→3′)	
	Forward	Reverse
*Gapdh*	AAGGTCATCCCAGAGCTGAA	AGGAGACAACCTGGTCCTCA
*Drd2*	TGAGGACATGAAACTCTGCA	CTGGTGCTTGACAGCATCTC
*Dat*	ATGTGGTCGTGGTCAGCATT	CTGGCAGGCTGCAGAACTTA
*Th*	TACTGGTTCACTGTGGAGTTT	TCTCCATAGGAAGACAGCAG
*Ache*	GCCTGAACCTGAAGCCCTTA	CTCGTCCAGAGTATCGGTGG
*Bdnf*	GCGCCCATGAAAGAAGTAAA	TCGTCAGACCTCTCGAACCT
*Nrxn1*	GCCTACACCTCTATGCATCT	CACTGATTGTCGTTGAGTGG
*Ncx2*	AGTGGATGATGAAGAGTATGAGAAGAAG	TTGGTTGAGTAGCAGAGCTGAGA
*Arc*	AAGTGCCGAGCTGAGATGC	CGACCTGTGCAACCCTTTC
*c-Fos*	TACTACCATTCCCCAGCCGA	GCTGTCACCGTGGGGATAAA
*Egr1*	TATGCTTGCCCTGTCGAGTC	GGATGTGGGTGGTAAGGTGG
*Avp*	ATCTGCTGCAGCGACGAGAGCT	AGAATCCACGGACTCCCGTGT
*Oxt*	CCTACAGCGGATCTCAGACTGA	TCAGAGCCAGTAAGCCAAGCA
*iNos*	TGACGGCAAACATGACTTCAG	GCCATCGGGCATCTGGTAG
*Iba1*	ATTTGCAGGGAGGAAAAGCTT	TGATCCCCTCCAGCCTCTCT
*Gfap*	AACCGCATCACCATTCCTGATA	TTTTGCCCCCTCGGATCT
*Tnf-α*	TCAAACCCTGGTATGAGCCC	ACCCATTCCCTTCACAGAGC
*Olig2*	AAGATCAACAGCCGCGAAC	AGTCGCTTCATCTCCTCCAG
*I* *l* *-6*	CCACGGCCTTCCCTACTTC	TTGGGAGTGGTATCCTCTGTGA
*I* *l* *-10*	GATGCCCCAGGCAGAGAA	CACCCAGGGAATTCAAATGC

## Data Availability

The datasets generated during and/or analyzed during the current study are available from the corresponding author on reasonable request.

## Supplementary Materials

The following supporting information can be downloaded at: https://www.mdpi.com/article/10.3390/ijms27073299/s1.

## Abbreviations

*Ache*	Acetylcholinesterase
*Avp*	Arginine vasopressin
*Bdnf*	Brain-derived neurotrophic factor
BPA	Bisphenol A
BPF	Bisphenol F
*c-fos*	fos proto-oncogene
CCK−8	Cell Counting Kit-8
*Dat*	Dopamine transporter
DNT	Developmental neurotoxicity
DOHaD	Developmental origins of health and disease
*Drd2*	Dopamine receptor D2
E	Embryonic day
EDC	Endocrine-disrupting chemical
*Gapdh*	Glyceraldehyde 3-phosphate dehydrogenase
*Gfap*	Glial fibrillary acidic protein
GFP	Green fluorescent protein
*Iba1*	Ionized calcium-binding adapter molecule 1
*Il-6*	Interleukin 6
*Il-10*	Interleukin 10
*iNos*	Inducible nitric oxide synthase
mESCs	Mouse embryonic stem cells
mLIF	Mouse leukemia inhibitory factor
NEAA	Non-essential amino acids
*Nrxn1*	Neurexin 1
*Olig2*	Oligodendrocyte transcription factor 2
*Oxt*	Oxytocin
PND	Postnatal day
*Th*	Tyrosine hydroxylase
TMBPF	Tetramethyl bisphenol F
*Tnf-α*	Tumor necrosis factor alpha
