# Supplementary material for: Effects of Maternal Tetramethyl Bisphenol F Exposure on Neurodevelopment and Behavior in Mouse Offspring
Source: Int J Mol Sci. 2026 Apr 5;27(7):3299. doi: 10.3390/ijms27073299 (PMC13072762; doi:10.3390/ijms27073299)
Supplement: Supplementary file 1 [file ijms-27-03299-s001.zip › ijms-4224117-supplementary.pdf]

**Supplementary table S1.** Statistical analysis of behavioral data

| **Group** | Treatment (Dam) | Dam no.* | Female offspring (n) | Male offspring (n) | Total (n) |
|---|---|---|---|---|---|
| Vehicle | Vehicle - Corn oil, 10 ml/kg/day (S.C.) | 1 | 5 | 3 | 8 |
| | | 3 | 3 | 4 | 7 |
| | | 5 | 4 | 3 | 7 |
| | | 7 | 2 | 4 | 6 |
| | Total (n) | 4 | 14 | 14 | 28 |
| TMBPF | TMBPF - 5.6 mg/kg/day in vehicle (S.C.) | 2 | 5 | 3 | 8 |
| | | 4 | 4 | 3 | 7 |
| | | 6 | 5 | 3 | 8 |
| | Total (n) | 3 | 14 | 9 | 23 |

* Dam numbers indicate individual pregnant females, and offspring counts represent the number of pups derived from each dam.

**Supplementary table S2.** Statistical analysis of behavioral data

| Test | Measurement | | Mean ± SEM values | Statistical test | | |
|---|---|---|---|---|---|---|
| | | | | Test | Test statistic value | P value |
| Open field | Distance traveled (cm) | | F VE = 3439.53 ± 301.82<br>F TMBPF = 1520.87 ± 261.54 | Unpaired t test | $T_{26}$ = 4.890 | <0.0001 |
| | | | M VE = 2830.97 ± 290.98<br>M TMBPF = 1293.41 ± 350.50 | | $T_{21}$ = 3.349 | 0.0030 |
| | Velocity (cm/s) | | F VE = 11.61 ± 1.03<br>F TMBPF = 7.48 ± 0.62 | | $T_{26}$ = 3.442 | 0.0020 |
| | | | M VE = 15.03 ± 5.65<br>M TMBPF = 7.88 ± 0.67 | | $T_{21}$ = 1.004 | 0.3270 |
| | Time in center (s) | | F VE = 19.60 ± 4.76<br>F TMBPF = 18.10 ± 3.40 | | $T_{26}$ = 0.257 | 0.7996 |
| | | | M VE = 15.84 ± 2.08<br>M TMBPF = 15.53 ± 4.78 | | $T_{21}$ = 0.058 | 0.9546 |
| | Number of entries into center | | F VE = 10.43 ± 1.10<br>F TMBPF = 7.57 ± 1.32 | | $T_{26}$ = 1.664 | 0.1082 |
| | | | M VE = 8.00 ± 0.50<br>M TMBPF = 6.89 ± 2.36 | | $T_{21}$ = 0.564 | 0.5789 |
| Nest building | Nesting score | | F VE = 4.64 ± 0.25<br>F TMBPF = 4.07 ± 0.32 | Unpaired t test | $T_{26}$ = 1.404 | 0.1721 |
| | | | M VE = 4.64 ± 0.25<br>M TMBPF = 4.22 ± 0.28 | | $T_{21}$ = 1.102 | 0.2831 |
| Forced swimming | Immobility time (s) | | F VE = 260.39 ± 5.26<br>F TMBPF = 277.09 ± 3.37 | Unpaired t test | $T_{26}$ = 2.674 | 0.0551 |
| | | | M VE = 276.31 ± 4.32<br>M TMBPF = 282.71 ± 4.34 | | $T_{21}$ = 0.957 | 0.3495 |
| Tail suspension | Immobility time (s) | | F VE = 212.57 ± 16.18<br>F TMBPF = 238.15 ± 7.79 | Unpaired t test | $T_{26}$ = 1.142 | 0.2668 |
| | | | M VE = 171.53 ± 18.92<br>M TMBPF = 194.22 ± 23.78 | | $T_{21}$ = 0.748 | 0.4628 |
| Morris water maze | Escape latency (s) | Day1 | F VE = 47.40 ± 3.15<br>F TMBPF = 40.60 ± 4.09 | Two-way ANOVA with Bonferroni's multiple comparisons test | $T_{26}$ = 1.493 | 0.6894 |
| | | | M VE = 47.42 ± 3.15<br>M TMBPF = 40.89 ± 4.63 | | $T_{21}$ = 1.229 | >0.9999 |
| | | Day2 | F VE = 33.00 ± 4.28<br>F TMBPF = 34.40 ± 3.58 | | $T_{26}$ = 0.307 | >0.9999 |
| | | | M VE = 33.03 ± 4.29<br>M TMBPF = 35.33 ± 3.47 | | $T_{21}$ = 0.433 | >0.9999 |
| | | Day3 | F VE = 19.50 ± 1.84<br>F TMBPF = 33.70 ± 3.53 | | $T_{26}$ = 3.118 | 0.0112 |
| | | | M VE = 19.45 ± 1.83<br>M TMBPF = 30.00 ± 3.91 | | $T_{21}$ = 1.986 | 0.2482 |
| | | Day4 | F VE = 19.10 ± 2.78<br>F TMBPF = 24.20 ± 2.49 | | $T_{26}$ = 1.120 | >0.9999 |
| | | | M VE = 19.08 ± 2.79<br>M TMBPF = 33.17 ± 4.47 | | $T_{21}$ = 2.652 | 0.0462 |
| | | Day5 | F VE = 15.30 ± 2.89<br>F TMBPF = 19.40 ± 2.81 | | $T_{26}$ = 0.900 | >0.9999 |
| | | | M VE = 15.27 ± 2.88<br>M TMBPF = 19.94 ± 6.01 | | $T_{21}$ = 0.879 | >0.9999 |
| | Platform crossing | | F VE = 2.91 ± 0.22<br>F TMBPF = 1.45 ± 0.32 | Unpaired t test | $T_{26}$ = 4.157 | 0.0003 |

| | | | | | | |
|---|---|---|---|---|---|---|
| | | | M VE = 3.57 ± 0.41<br>M TMBPF = 1.82 ± 0.44 | | $T_{21}$ = 2.815 | 0.0104 |
| | Platform time (s) | | F VE = 1.61 ± 0.34<br>F TMBPF = 0.76 ± 0.18 | | $T_{26}$ = 2.301 | 0.0297 |
| | | | M VE = 2.16 ± 0.31<br>M TMBPF = 0.79 ± 0.14 | | $T_{21}$ = 3.398 | 0.0027 |
| Novel object recognition | Recognition index (%) | | F VE: Familiar = 41.77 ± 2.71<br>F VE: Novel = 58.23 ± 2.71 | Unpaired t test | $T_{26}$ = 4.292 | 0.0002 |
| | | | F TMBPF: Familiar = 48.36 ± 4.31<br>F TMBPF: Novel = 51.64 ± 4.31 | | $T_{26}$ = 0.5371 | 0.5962 |
| | | | M VE: Familiar = 41.77 ± 2.71<br>M VE: Novel = 58.23 ± 2.71 | | $T_{26}$ = 4.520 | 0.0001 |
| | | | M TMBPF: Familiar = 45.71 ± 2.39<br>M TMBPF: Novel = 54.29 ± 2.39 | | $T_{14}$ = 2.536 | 0.0238 |
| | Total exploring time (s) | | F VE = 100.86 ± 6.49<br>F TMBPF = 55.64 ± 6.30 | | $T_{26}$ =4.985 | <0.0001 |
| | | | M VE = 90.29 ± 9.29<br>M TMBPF = 53.03 ± 12.39 | | $T_{20}$ = 2.443 | 0.0235 |
| 3-chamber | Preference index | Social ability | F VE = 1.36 ± 0.20<br>F TMBPF = 1.14 ± 0.22 | Unpaired t test | $T_{26}$ = 0.751 | 0.4606 |
| | | | M VE = 1.42 ± 0.21<br>M TMBPF = 1.10 ± 0.24 | | $T_{20}$ = 0.950 | 0.3533 |
| | | Social novelty | F VE = 1.48 ± 0.26<br>F TMBPF = 1.14 ± 0.29 | | $T_{26}$ = 0.869 | 0.3928 |
| | | | M VE = 1.44 ± 0.23<br>M TMBPF = 2.15 ± 0.29 | | $T_{20}$ = 1.900 | 0.0720 |
| Social interaction | Number of sniffing events | | F VE = 28.86 ± 1.39<br>F TMBPF = 26.79 ± 1.64 | Unpaired t test | $T_{26}$ = 0.962 | 0.3450 |
| | | | M VE = 36.36 ± 2.90<br>M TMBPF = 29.22 ± 1.77 | | $T_{21}$ = 1.827 | 0.0820 |
| | Number of anogenital events | | F VE = 27.71 ± 2.04<br>F TMBPF = 18.36 ± 1.80 | | $T_{26}$ = 3.441 | 0.0020 |
| | | | M VE = 28.57 ± 2.20<br>M TMBPF = 21.22 ± 2.34 | | $T_{21}$ = 2.204 | 0.0388 |
| | Number of following events | | F VE = 9.71 ± 1.59<br>F TMBPF = 5.43 ± 0.94 | | $T_{26}$ = 2.197 | 0.0368 |
| | | | M VE = 11.07 ± 1.46<br>M TMBPF = 6.56 ± 1.18 | | $T_{21}$ = 2.190 | 0.0399 |

F: Female; M: Male; VE: Vehicle
